# The promise and limitations of artificial intelligence in musculoskeletal imaging

**DOI:** 10.3389/fradi.2023.1242902

**Published:** 2023-08-07

**Authors:** Patrick Debs, Laura M. Fayad

**Affiliations:** ^1^ The Russell H. Morgan Department of Radiology & Radiological Science, The Johns Hopkins Medical Institutions, Baltimore, MD, United States; ^2^ Department of Orthopaedic Surgery, Johns Hopkins University School of Medicine, Baltimore, MD, United States; ^3^ Department of Oncology, Johns Hopkins University School of Medicine, Baltimore, MD, United States

**Keywords:** artificial intelligence, machine learning, neural networks, musculoskeletal imaging, image interpretation, automation

## Abstract

With the recent developments in deep learning and the rapid growth of convolutional neural networks, artificial intelligence has shown promise as a tool that can transform several aspects of the musculoskeletal imaging cycle. Its applications can involve both interpretive and non-interpretive tasks such as the ordering of imaging, scheduling, protocoling, image acquisition, report generation and communication of findings. However, artificial intelligence tools still face a number of challenges that can hinder effective implementation into clinical practice. The purpose of this review is to explore both the successes and limitations of artificial intelligence applications throughout the muscuskeletal imaging cycle and to highlight how these applications can help enhance the service radiologists deliver to their patients, resulting in increased efficiency as well as improved patient and provider satisfaction.

## Introduction

Radiological imaging has come to play a central role in the diagnosis and management of different muscuskeletal (MSK) disorders, and both technological improvements and increased access to medical imaging have led to a rise in the utilization of common MSK imaging modalities (1, 2). As such, there is a growing need for technical innovations that can help optimize workflow and increase productivity, especially in radiology practices that are witnessing higher volumes of increasingly complex cases (3).

**Figure 1:**
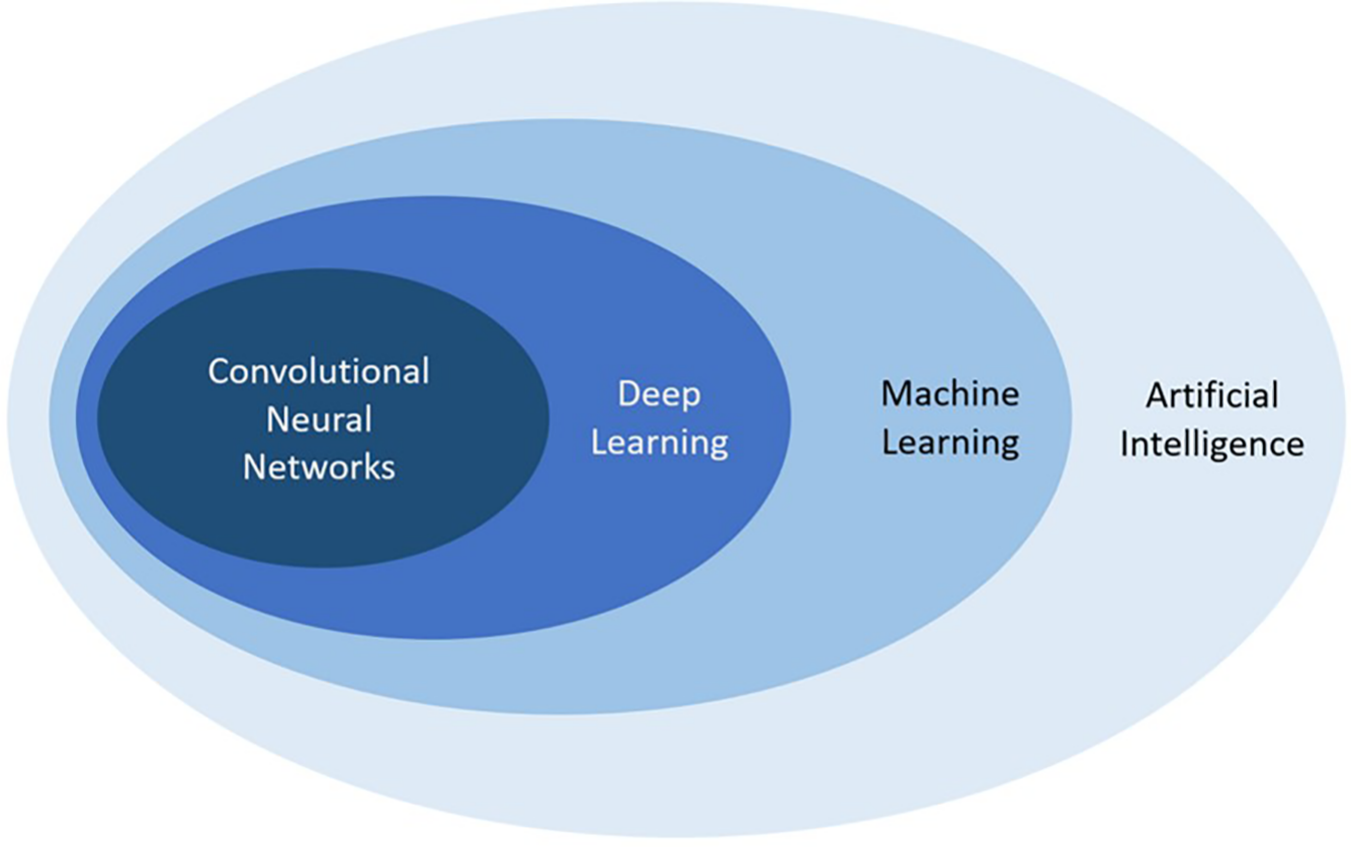
Schematic representation demonstrating the relationship between artificial intelligence, machine learning, deep learning, and convolutional neural networks, all subfields of each other.

Artificial intelligence (AI), or the development of computer systems that can mimic human intelligence when performing human tasks, is rapidly expanding in the field of diagnostic imaging and could potentially help improve workflow efficiency (4). AI is a broad term that encompasses numerous techniques, and recent advances in the field have transformed this technology into a powerful tool with several promising applications. Nested within AI is machine learning (ML), a subfield that gives computers the ability to learn and adapt by drawing inferences from patterns in data without following explicit instructions (5). ML uses observations from data to create algorithms and subsequently makes use of these algorithms to determine future output, with the goal of designing a system that can automatically learn without any human intervention. Deep learning (DL) is an even more specialized subfield within ML that uses multiple processing layers to progressively extract higher-level features from raw input presented in the form of large datasets, and the recent development of DL with convolutional neural networks (CNN) is an important technological advancement apt at solving image-based problems with reportedly outstanding performance in several key aspects of medical imaging (Figure 1) (6–8). CNNs are widely used in computer vision; they represent feedforward neural networks with multiple layers of non-linear transformations between inputs and outputs and can be programmed to classify an image or objects according to their features (output) by means of a training dataset with numerous images or objects (input) (4).

For AI models to be developed, large data sets with high-quality images and annotations are needed for both training of a model and validation of its performance, and, given that developers are usually not located within medical practices or hospital systems and therefore do not have access to such data, image sharing between the two becomes necessary. This multi-step process requires collaboration between clinicians and developers and, following approval from the responsible ethical committees, begins with image de-identification, storage, and resampling of resolutions (9). Images must then be appropriately labeled with ground truth definitions, and, depending on the outcome of interest, this can involve several different steps such as manual labeling of images, data extraction from medical charts and pathology reports, and detection of imaging findings from radiology reports or by radiologists' re-review of the imaging findings (9). Typically, data sets used for training are larger than data sets used for validation and testing, and, although logistically challenging in many cases, images should ideally be obtained from multiple diverse sources to increase representation of different populations and ensure generalizability of the model's performance (9).

**Figure 2:**
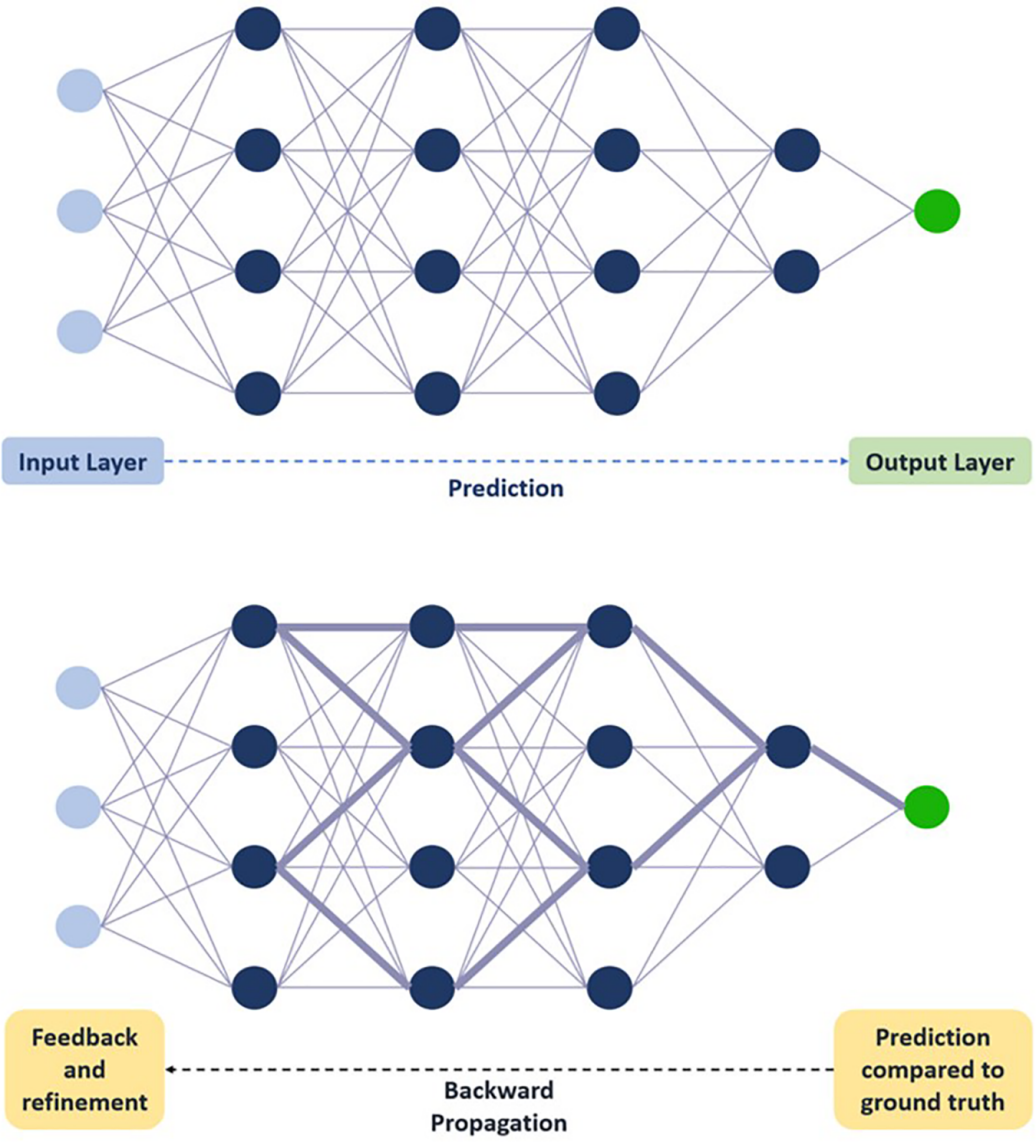
Schematic representation of deep neural network training. Training data is fed to the network at the initial input layer and propagated through subsequent layers for a prediction to be made at the final output layer. Prediction is compared to ground truth, and feedback through backward propagation leads to progressive refinement of weights. Circles represent nodes. Lines connecting circles represent weighted connections, with thickness correlating with weight magnitude. Dashed arrow represents flow of information through network.

To understand how images are used for training AI and DL models, it is important to understand the architecture of the neural networks often employed in such models. The basic building block of a deep neural network is a node, which can be considered analogous to a neuron, and one neural network is comprised of several weighted nodes arranged into layers and connected through weighted connections (10). Training data is fed to a network at an initial input layer and then propagated throughout all layers of the model: each layer performs both linear (e.g., weighted additions) and non-linear (e.g., thresholding) mathematical computations from input received from the previous layer and feeds the output to the next layer, which then performs the same computations until one final output layer is reached (10). The model then provides a prediction, which is compared to the ground truth label previously assigned. Discrepancies between the two are fed back into the network through backward propagation and gradient descent: nodal weights and connections are adjusted accordingly, and the model is refined with every data point from the training set (Figure 2). Once the model is sufficiently refined, a validation set is typically used to evaluate the model's generalizability and further refine predictions, after which the model is then tested using a final test set with unseen data to simulate and assess real-life performance (10). The size of the data sets needed for training, validation, and testing can vary depending on the outcome and/or the targeted population (with larger sets needed for populations with more diversity and heterogeneity) but generally follows a ratio of 80:10:10 or 70:15:15, respectively (9).

With its rapid and exponential growth, AI has the potential to significantly strengthen several steps of the MSK imaging value chain and offer applications that extend beyond imaging interpretation to assist with non-interpretive tasks such as patient scheduling, optimal protocoling, image acquisition, and data sharing (11). AI can theoretically improve an MSK radiologist's ability to respond to the increasing workload of high-volume practices and continue delivering high-quality care by allotting more time for demanding tasks and minimizing time spent on more routine and less complex functions. However, AI is not without its pitfalls, and overutilization of this resource can pose multiple problems relating to medical errors, bias and inequality, data availability, and privacy concerns (12). The purpose of this review is to highlight the different applications AI is presently offering or can potentially offer throughout the MSK radiology imaging cycle and to discuss risks, limitations, and future directions of this important technology.

## Prominent AI applications

### Image appropriateness and protocoling

The first step in the MSK imaging process is to order the appropriate imaging test, the responsibility of which falls on the referring clinician or provider confronted with a wide range of available modalities. AI, and ML in particular, could help facilitate the process: ML algorithms could be used to generate holistic clinical decision support systems that can consider various aspects from a patient's medical chart such as symptomatology, laboratory test results, physical examination findings, and previous imaging to recommend the modality best suited to address the clinical query in question (13, 14).

Protocoling comes next, and once an imaging modality is chosen, the MSK radiologist or trainee is usually responsible for ensuring that scans are performed correctly. Choosing the right protocol is crucial to reaching a proper diagnosis and optimizing patient care but can prove arduous and time-consuming for the radiologist tasked with several other responsibilities; as such, several recent studies have looked into how DL can be of assistance. Lee assessed the feasibility of using short-text classification to develop a CNN classifier capable of determining whether MRI scans should be completed following a routine or tumor protocol and, after comparing CNN-derived protocols to those determined by MSK radiologists, reported an area under the curve (AUC) of 0.977 and an accuracy of 94.2% (15). Similarly, Trivedi et al. developed and validated a DL-based natural language classifier capable of automatically determining the need for intravenous contrast for MSK-specific MRI protocols based on the free-text clinical indication of the study and reported up to 90% agreement with human-based decisions (16). Although these studies show promising results, MSK imaging protocols are complex and diverse, given that MSK as a field encompasses localized and systemic diseases from neck to toe. More investigations could potentially explore the use of other composite classifiers such as medical history, prior imaging protocols, scanner-specific data, contrast information, and radiation exposure to help with protocoling decisions (13).

### Scheduling

Given the rise in the use of medical imaging, adherence to set schedules has become more important for radiology practices, especially in the MSK setting where advanced and sometimes lengthy examinations such as MRI and CT are frequently used. No-shows or appointment cancellations can be a significant burden on practices and also represent missed opportunities for other patients to be scanned (17). There has been a growing interest in how AI can help optimize scheduling in various medical practices, and ML algorithms with predictive frameworks have been successfully used to predict missed appointments in diabetes clinics as well as urban, academic, and underserved settings (18, 19). Various ML predictive models have also been used to predict imaging no-shows effectively (20, 21), and Chong et al. demonstrated how using a pre-trained CNN with a predictive framework to predict MRI no-shows and accordingly send out proactive reminders to patients resulted in a reduced appointment no-show rate from 19.3% to 15.9% (22). ML could also help maximize patient throughput; Muelly et al. developed a feed-forward neural network that can make use of patient demographics and dynamic block lengths to estimate average MRI scan durations, resulting in decreased wait times, improved patient satisfaction, and optimized schedule fill rates (23).

### Image acquisition

#### Magnetic resonance imaging acquisition

Given the critical need but lengthy nature of MRI scans in MSK imaging, there has always been an interest in reducing MRI acquisition times in order to decrease patient discomfort and improve scanner efficiency. Previous attempts at MRI acceleration focused on parallel imaging and compressed sensing, both of which operate by subsampling k-space and reducing the number of phase-encoding lines acquired during a scan, ultimately resulting in less data being collected (24, 25). While efficient, these two techniques suffer from reduced image quality and increased artifacts in the reconstructed images, leading to less diagnostic imaging. ML has been proposed as a possible solution that can help mitigate the limitations of accelerated imaging by using subsampled k-space data to generate up-sampled high-resolution output images comparable to images generated from otherwise fully sampled k-space data (26). Using high quality MR images, Wang et al. trained a CNN to restore fine structural details on brain images obtained from zero-filled k-space data and were able to generate images of diagnostic quality comparable to images from a fully sampled k-space but with a fivefold increase in acquisition speed (27). Hammernik et al. were able to achieve a fourfold increase in knee MRI acquisition speed by using a DL technique that created high-quality reconstructions of under-sampled data (28), while Chaudhari et al. successfully made use of a CNN to output thin-slice knee images from thicker slices, thereby improving spatial resolution and image quality (29). Similarly, Wu et al. developed an eightfold-accelerated DL model capable of up-sampling sparsely sampled MRI data to output images with minimal artifacts and a permissible signal-to-noise ratio (30). In one study by Roh et al., DL-accelerated turbo spin echo sequences were assessed for their ability to depict acute fractures of the radius in patients wearing a splint and were shown to be effective for both increasing acquisition speed by a factor of 2 as well as improving image quality when compared to standard sequences (31). Studies are still ongoing, with AI-driven 10-fold accelerated MRI increasingly becoming within reach (32) and other exciting ML applications being explored such as the production of MR images from CT images (33) and the post-processing of a single MRI acquisition to obtain other planes and tissue weightings (34). One such advance in MSK imaging is the synthetic construction of fat-suppressed imaging from non-fat-suppressed imaging (35).

#### Computed tomography

Unlike MRI, CT exposes patients to ionizing radiation, and ML has shown promise as a tool that can help reduce the radiation dose of a CT scan while maintaining a high quality of images (36). The premise is similar to ML applications for MRI acquisitions, whereby the goal is to reconstruct images of diagnostic quality using lower-quality source data or reduced quantities of source data. Cross et al. demonstrated how CT images acquired at a low radiation dose and reconstructed in part using an artificial neural network were found to be similar to or improved compared to images obtained using standard radiation doses by more than 90% of the readers in the study (37). Other AI developments can also help enhance image quality by decreasing artifacts related to different factors, as demonstrated in the study by Zhang and Yu where a CNN trained to merge original- and corrected-image data was capable of suppressing metal artifacts and preserving anatomical structural integrity near metallic implants (38).

### Image presentation

In radiology practices that use a Picture Archiving and Communication System (PACS), radiologists often spend a considerable amount of time manipulating image displays and toggling between sequences and viewing panes to display different imaging features in several anatomic planes. This is known as the hanging protocol and constitutes another venue that can be enhanced through the use of AI to afford radiologists more productivity and efficiency. A study by Kitamura showed how ML techniques using DenseNet-based neural network models can successfully optimize hanging protocols of lumbar spine x-rays by considering several parameters such as dynamic position and rotation correction (39). Moreover, one PACS vendor is currently using ML-based algorithms to learn a radiologist's preferences when viewing examinations, record orientations of the sequences most commonly used, suggest displays for future similar studies, and incorporate adaptations following every correction, all in an effort to improve the workflow in the reading room (40).

### Image interpretation

Although AI can assist MSK radiologists with several steps of the imaging cycle, it is AI's ability to help with image interpretation, arguably a radiologist's most important responsibility, that has garnered the most attention in recent years. The next section discusses different ways AI and ML can help radiologists with MSK imaging interpretations to diagnose different conditions with greater efficiency. Table 1 provides a summary of AI tools with such applications.

**Table 1:** AI tools in musculoskeletal imaging.

Publication	Application in musculoskeletal imaging	Algorithm type	System performance
Chung et al. (41)	Proximal humerus fracture detection	CNN	Sensitivity/specificity: 0.99/0.97 Accuracy: 96%
Olczak et al. (42)	Ankle, wrist and hand radiographic fracture detection	VGG 16-layer CNN	Accuracy: 83%
Yu et al. (43)	Hip fracture detection	CNN	Sensitivity/specificity: 97.1%/96.7%
Tomita et al. (44)	Osteoporotic vertebral fracture detection	CNN	Accuracy: 89.2%
Cheng et al. (45)	Hip fracture detection	CNN	Accuracy: 91% Sensitivity: 98%
Rajpurkar et al. (46)	Radiographic abnormality detection	169-layer DenseNet	Sensitivity/specificity: 81.5%/88.7%
Xue et al. (47)	Hip osteoarthritis detection	CNN	Sensitivity/specificity: 95.0%/90.7% Accuracy: 92.8%
Tiulpin et al. (48)	Knee osteoarthritis detection	Deep Siamese CNN	Accuracy: 66.71%
Antony et al. (49)	Knee osteoarthritis severity grading	CNN	Variable
Pedoia at el. (50)	Osteoarthritis cartilage degenerative change detection and staging	CNN	Accuracy: 80.74%, 78.02%, and 75.00% for normal, small, and complex large lesions, respectively
Liu et al. (51)	Knee cartilage lesion detection	CNN	Sensitivity/specificity: 84.1%/85.2% 80.5%/87.9%
Halabi et al. (52) Model by Cicero and Bilbily	Bone age detection	Google Inception V3 network	Mean absolute difference from ground truth: 4.265 months
Tajmir et al. (53)	Bone age detection	CNN	Accuracy: 98.6% within one year
Kim et al. (54)	Computer-assisted bone age detection	Greulich-Pyle method-based DL	69.5% correlation rate with reference bone age
Thodberg et al. (55) Martin et al. (56) Maratova et al. (57)	Bone age detection software validation	BoneXpert	Variable
Yang et al. (58)	Bone strength prediction	Scaling index method	Root mean square error: 0.869 ± 0.121 Coefficient of determination R^2^: 0.68 ± 0.079
Huber et al. (59)	Bone biomechanical property detection	Scaling index method	Root mean square error: 1.021
Deniz et al. (60)	Proximal femur segmentation	CNN	Dice similarity score: 0.95 ± 0.02
Lee et al. (61)	Osteoporosis detection in panoramic radiographs	CNN	AUC values: 0.9763, 0.9991 and 0.9987
Pan et al. (62)	Osteoporosis screening using low-dose chest CT	3D U-net CNN	AUC: 0.927 for detecting osteoporosis and 0.942 for distinguishing low BMD
Jimenez-Pastor et al. (63)	Vertebrae localization and identification	Decision forests and image-based refinement	Identification rate: 79.6% for the thoracic and of 74.8% for the lumbar region
Lessmann et al. (64)	Vertebrae segmentation and identification	CNN	Dice score: 94.9 ± 2.1% for segmentation Accuracy: 93% for identification
Wimmer et al. (65)	Vertebral body and intervertebral disc localization and labeling	CNN	Detection rate: 93.6%
Jamaludin et al. (66)	Lumbar spine MRI radiological feature detection	CNN	Accuracy: 95.6% for disc detection and labeling
Lu et al. (67)	Automating lumbar vertebral segmentation, disc-level designation, and spinal stenosis grading	U-net CNN	Variable
Han et al. (68)	Spinal structure segmentation	Generative Adversarial Network	Accuracy:96.2% Dice coefficient: 87.1%, Sensitivity/specificity: 89.1%/86.0%
Pan et al. (69)	Radiographic Cobb angle measurement and scoliosis detection	Mask R-CNN	Sensitivity/specificity: 89.59%/70.37%
Weng et al. (70)	Radiographic sagittal vertical axis measurement	ResUNet CNN	Median absolute error: 1.183 ± 0.166 mm
Kim et al. (71)	Differentiating between tuberculous and pyogenic spondylitis	CNN	AUC: 0.802
Acar et al. (72)	Differentiating metastatic and completely responded sclerotic bone lesion in prostate cancer	Textural analysis, support vector machine, K-nearest neighbor, ensemble classifier	Variable
Lang et al. (73)	Differentiating spinal metastases origin cancer	Radiomics, CNN, CLSTM	Accuracy: 0.71 for radiomics, 0.71 for CNN, 8.81 for CLSTM
Malinauskaite et al. (74)	Differentiating soft-tissue lipoma and liposarcoma	Radiomics and ML classifier	AUC: 0.926
Zhang et al. (75)	MRI histopathological grading of soft tissue sarcomas	Radiomics, random forests, k-nearest neighbor, support vector machine	Accuracy: 0.88
He et al. (76)	Predicting recurrence of giant cell bone tumors	CNN and CNN regression models	Accuracy: 75.5% and 78.6%
Blackledge et al. (77)	Segmenting and evaluating soft tissue sarcomas after radiotherapy	Logistic regression, support vector machine, random forest, k-nearest neighbor, kernel density estimation, Naïve-Bayes, 20-node, three-layer, fully-connected neural network	Variable
Bien et al. (78)	Detecting various knee abnormalities	CNN	Variable
Liu et al. (79)	Diagnosing anterior cruciate ligament tears	CNN	Sensitivity/specificity: 96%/96% AUC: 0.98
Ma et al. (80)	Diagnosing meniscal injuries of the knee	CNN	Average accuracy: 89.8%
Chang et al. (81)	Detecting complete anterior cruciate ligament tears	CNN	Accuracy: 96%
Couteaux et al. (82)	Detecting meniscal tears of the knee	Region-based CNN	AUC: 0.906
Roblot et al. (83)	Detecting meniscal tears of the knee	CNNs	AUC: 0.90
Liu et al. (84)	Segmenting knee cartilage and bone	CNNs	Variable
Norman et al. (85)	Segmenting knee cartilage and menisci	CNNs	Variable dice scores, ranging between 0.770 and 0.878 for cartilage and 0.809 and 0.753 for menisci
Balsiger et al. (86)	Peripheral nerve segmentation	CNN	Dice scores of 0.859 and 0.719
Kemnitz et al. (87)	Thigh muscle and adipose tissue segmentation	U-Net CNN	Dice score: 0.96
Yin et al. (88)	Differentiating sacral chordoma from sacral giant cell tumor	Radiomics ML classifiers	Variable
Gitto et al. (89)	Classifying deep-seated lipomas and atypical lipomatous tumors of the extremities	Radiomics-based ML	Sensitivity/specificity: 92%/33%
Pfeil et al. (90)	Joint-space analysis for rheumatoid arthritis detection	Computer-aided joint space analysis	Variable
Langs et al. (91)	Erosion spotting and visualization in rheumatoid arthritis	Generative appearance model	Sensitivity/specificity: 85%/84% AUC: 0.92
Liu et al. (92)	Epidural mass detection	Gaussian Mixture Model	Accuracy: 82%
Stotter et al. (93)	Radiographic measurements of the pelvis and hip	CNN	Variable
Etli et al. (94)	Sex estimation from sacrum and coccyx	Univariate discriminant analysis, linear discriminant function analysis, stepwise discriminant function analysis, multilayer perceptron neural networks	Accuracy: 67.1% for univariate discriminant analysis, 82.5% for linear discriminant function analysis, 78.8% for stepwise discriminant function analysis, and 86.3% for multilayer perceptron neural networks
Yune et al. (95)	Predicting sex from hand radiographs	CNN	Agreement with phenotypic sex: 77.8%
Bowness et al. (96)	Identifying anatomical structures on ultrasound	U-Net CNN	mean highlighting scores ranging from 7.87/10 to 8.69/10

AUC, area under the curve; BMD, bone mineral density; CLSTM, convolutional long short-term memory; CNN, convolutional neural network; CT, computed tomography; DL, deep learning; ML, machine learning; MRI, magnetic resonance imaging; VGG, visual geometry group.

#### Fractures

Automated fracture detection using AI can be helpful not only to radiologists but also to other clinicians (such as overnight emergency department personnel) who might not always have access to radiology services and would sometimes have to rely on their own preliminary fracture diagnosis. DL techniques have been gaining increasing attention over the past few years in their ability to detect fractures on images, as this can increase diagnostic reliability and reduce the rate of medical errors.

Some studies have shown that CNNs can outperform orthopedic surgeons when it comes to the detection of upper limb and ankle fractures on radiographs (41, 42). Additionally, multiple studies have shown promise when assessing the competence of AI in detecting both axial and appendicular skeletal fractures on radiographic and CT images (26, 43, 44, 97, 98), with one CNN model by Cheng et al. achieving an AUC of 0.98 and an accuracy of 91% for radiographic hip fracture detection (45). Rajpurkar et al. trained a 169-layer DenseNet baseline model to detect and localize fractures using a large dataset of MSK radiographs containing 40,561 manually-labeled images; when tested on a set of 207 studies, the model successfully detected finger and wrist abnormalities with an AUC of 0.929 but was less competent at detecting abnormalities of the shoulder, humerus, elbow, forearm, and hand (46). Since then, their large dataset was made publicly available under the name MURA to encourage public submissions and improve fracture detection rates of the original study (99). With all the collective efforts being made to improve AI-assisted fracture detection, models are no longer just objects of research studies but have been implemented into clinical practice. Presently, Gleamer BoneView (Gleamer, Paris, France) is an FDA-approved commercially available software that can help detect fractures on radiographs and is the only AI fracture detection software to have FDA clearance for use in both adults and pediatric patients over two years of age (100).

However, despite all these promising applications, AI-assisted fracture detection still has a key limitation: each CNN model must be specifically trained on the body part being assessed using large numbers of properly labeled images, whereas humans can transfer their knowledge of one body part to another. Moreover, models can be less reliable when trying to detect less obvious fractures such as a non-displaced femoral neck fracture (98), and most models report the output in a binary fashion (fracture present or not present) without providing an in depth description of the lesion or other related findings.

### Osteoarthritis

Several studies have looked into how AI can assist radiologists in evaluating images for the presence and grading of osteoarthritis. Xue et al. fine-tuned a CNN model using a set of 420 hip radiographs to detect hip osteoarthritis using a binary system and reported a performance akin to that of a radiologist with ten years of experience (47). Tiulpin et al. took advantage of the large publicly available Osteoarthritis Initiative (OAI) and Multicenter Osteoarthritis Study (MOST) datasets to train and test a CNN model to automatically score knee osteoarthritis severity using to the Kellgren-Lawrence grading scale and reported promising results with an AUC of 0.80 (48). Interestingly, the probability distribution of KL grades was also reported to show when predicted probabilities may be comparable across two contiguous grades, rendering the model's performance more illustrative of real-life practice where arthritis severity may represent the transition between two adjacent grades instead of being neatly tiered at one single level. This was also done in a study by Antony et al. where, in an attempt to circumvent the limitation of a finite and discreet scale, knee osteoarthritis grading was redefined as a regression with continuous variables (49). Although osteoarthritis assessment has been traditionally done using radiographs, AI can also augment the quantitative and qualitative assessment of cartilage on MRI to render the evaluation of osteoarthritis more accurate, and several studies have worked on developing models capable of successfully detecting cartilage lesions and staging cartilage degenerative changes (50, 51).

#### Bone age

Radiographic assessment of bone age is important for pediatricians to assess the skeletal maturity and growth of a child, and efforts have been put into using AI to automate bone age assessment and avoid the use of the inflexible and error-prone traditional methods such as the Greulich-Pyle atlas and the Tanner-Whitehouse method (101, 102). The Radiological Society of North America Pediatric Bone Age Machine Learning Challenge freely provided ML developers with a dataset containing over 14,000 hand radiographs and used competitions to promote collaborative effort into designing tools competent at automating bone age assessment (52). With over 100 submissions, the winning algorithm was designed by the University of Toronto's Cicero and Bilbily who used Google's Inception V3 network for pixel information, concatenated the architecture with sex information, and added layers after concatenation for data augmentation (52). The ultimate goal would be to provide radiologists with a tool that can help them assess bone age rather than perform the task independently. Tajmir et al. revealed how radiologists assisted by AI software when assessing bone age perform better than an unaided AI model, a single radiologist working independently, and a group of expert radiologists working together (53). Moreover, Kim et al. showed how the use of AI software can reduce reading times by approximately 30%, from 1.8 to 1.38 min per study (54). Presently, BoneXpert is a commercially-available widely-used software developed by Visiana that provides automated bone assessment by delineating the distal epiphyses of several hand bones, with at least eight needed for computation (55). Using the Greulich and Pyle or Tanner-Whitehouse standards, skeletal maturity is assessed with a precision of 0.17 years, reportedly nearly three times better than human performance (14, 56, 57).

#### Bone fragility

Imaging is often used for the evaluation of osteoporosis, a bone disorder characterized by a decreased bone mineral density (BMD), as bone strength assessment is fundamental for clinical decision making and therapy monitoring. Several studies have coupled ML support vector machines with methods of evaluating trabecular bone microarchitecture to automate and improve quantitative bone imaging and assessment (58, 59). In one study, Yang et al. used DL algorithms to combine BMD data from dual-energy x-ray absorptiometry (DXA) with bone microarchitecture data from multi-detector CT in an attempt to predict proximal femur failure loads; analysis revealed that trabecular bone characterization and ML methods, when coupled with conventional DXA BMD data, can appreciably enhance biomechanical strength prediction (58). Huber et al. applied similar ML methods to predict proximal tibial trabecular bone strength using MRI data instead and concluded that combining ML techniques with data on bone structure can enhance MRI assessment of bone quality (59).

In the same vein, ML algorithms have been employed in an attempt to predict osteoporotic fractures from MRI data (103), with one study making use of a CNN to automate segmentation of the proximal femur and facilitate the measurement of bone quality on MRI (60). Research has also focused on developing tools that can offer opportunistic screening and assessment of bone fragility, with one study looking at a system that can evaluate bone quality on dental panoramic radiographs (61) and another describing a DL system that can measure BMD on low-dose chest CT performed for lung cancer screening (62). With all those recent developments, AI is showing promise as a tool that can help with osteoporosis diagnosis; however, further refinement of such models is still needed to better automate the objective assessment of osteoporosis, its progression, and its response to therapy (104).

#### Spine imaging

Given that MSK radiologists spend a considerable amount of time looking at spine imaging, efforts have been made to develop ML algorithms that can automate tasks related to spine imaging interpretation and decrease the amount of time needed to interpret individual scans (105). Multiple studies have presented AI tools that can successfully detect and label spinal vertebrae as well as intervertebral discs on MRI and CT images (63–65), obviating the need for human manual labeling and streamlining the review of images. Information from these models can be used to automate other processes, as demonstrated by Jamaludin et al. who, after presenting a model that could label vertebral bodies and intervertebral discs on MRI with a 95.6% accuracy, used a CNN to successfully provide radiologist-level assessment of several other findings such as disc narrowing, central canal stenosis, spondylolisthesis, and end plate defects (66).

In addition to that, researchers have focused on designing models that can automate segmentation of the vertebrae, with one study making use of a U-Net architecture to segment the six lumbar intervertebral disc levels (67) and another adopting an iterative instance approach whereby information on one segmented vertebra is used to iteratively detect the following one (64). In the former study, Lu et al. also trained their model to automate spinal and foraminal stenosis grading using a large dataset obtained from 4,075 patients and reported an accuracy of 80% for grading spinal stenosis and 78% for grading neural foraminal stenosis (67). To increase concordance between automated segmentation outputs and ground truth labels, generative adversarial networks have also been used, with one resultant model concurrently segmenting the neural foramen, the vertebral bodies, and intervertebral discs (68).

Advancements in this line of research are ongoing, supported by large publicly available datasets such as SpineWeb and the MICCAI 2018 Challenge on Automatic Intervertebral Disc Localization and Segmentation dataset. The Pulse platform (NuVasive, San Diego, California, USA) is a recent FDA-approved spinal surgical automation platform that combines multiple technologies to provide intraoperative assessment during spine surgeries and can help with tasks such as neuromonitoring of nerves, improvement of screw placement, and minimizing intraoperative radiation exposure (106). Other spine imaging applications could include automating radiographic measurements of spinal alignment (69, 70) and using CNNs to distinguish tuberculous from pyogenic spondylitis (71). However, despite the promising results of all these recent developments, further research is still needed, and studies are often hindered by several limitations such as the lack of a consistent gold standard for entities where radiologists may exhibit high variability in interpretation (107).

#### Muscuskeletal oncology

AI can potentially have several applications in MSK oncology and may be able to help radiologists detect metastatic bone lesions, determine their origin, and assess progression and treatment response. Using CT texture analysis, Acar et al. developed an ML model with an AUC reaching up to 0.76 when differentiating metastatic bone lesions from sclerotic bone lesions with complete response in patients with prostate cancer (72). To determine tumor origin on contrast-enhanced MRI, Lang et al. used DL methods and radiomics to devise a model that successfully differentiated between spinal metastatic lesions from the lung and other origin sites with a high accuracy reaching 0.81 (73). In addition, AI can potentially help with the assessment of primary musculoskeletal tumors. For example, two studies making use of ML techniques and radiomics demonstrated how lipoma and liposarcoma could be differentiated on MRI with expert-level performance (74) and how the histopathological grades of soft tissue sarcomas can be pre-operatively and non-invasively predicted on fat-suppressed T2-weighted imaging with an accuracy reaching 0.88 (75). AI might also serve other proposed roles, such as assisting clinicians in predicting tumor recurrence as well monitoring post-treatment tumor changes on imaging (76, 77).

#### Cruciate ligaments and menisci

Several studies have evaluated the performance of AI models when detecting meniscal injuries and ligamentous tears of the knee. Bien et al. trained a CNN model using a set of 1,130 training and 120 validation MRI exams to recognize meniscal and anterior cruciate ligament (ACL) tears, reporting an AUC of 0.847 for meniscal tears and an AUC of 0.937 for ACL tears on the internal validation set and 0.824 on the external validation set (78). Using arthroscopy as the reference, Liu et al. trained a CNN to isolate ACL lacerations with an AUC of 0.98 and a sensitivity of 96% (51, 79), and Ma et al. trained a CNN to diagnose meniscal injury, reporting a an accuracy of 85.6% for anterior horn injury detection and 92% for posterior horn injury detection, a performance comparable to a chief physician (80).

Isolation of individual joint structures might help enhance model performance, as demonstrated by Chang et al. who, after isolating the ACL on coronal proton density 2D MRI using CNN U-Net, subsequently used a CNN classifier to evaluate the isolated ACLs for the presence of pathology and reported an AUC of 0.97 and a sensitivity of 100% (81). When testing a CNN model for meniscal segmentation on fat-suppressed MRI sequences, Pedoia et al. reported a sensitivity reaching 90%, a specificity reaching 82%, and an AUC reaching 0.89 (50). Likewise, Couteaux et al., Roblot et al., and Lassau et al. all reported similar performances, with AUC values for meniscal tear detection reaching 0.9 in all three studies (82, 83, 108).

#### Quantitative analysis: segmentation and radiomics

Segmentation, or the process of delineating anatomic structures, can be time-consuming but is nevertheless important for evaluating the potential degeneration of or damage to segmented structures and the resultant decline in their functionality. Semi-automated segmentation software are currently being applied in clinical cardiac and prostate MRI, but such software make use of algorithms with manually designed hand-engineered features and thus require manual adjustments to the computer-generated contours (26). As such, interest has shifted to fully automating segmentation processes using CNN, which can have a profound impact on a radiologist's functionality and efficiency in the reading room. Performance of segmentation algorithms is often assessed with a dice coefficient to assess the similarity of a segmentation to its ground truth by reporting the percentage overlap between the two regions, and a dice score of 0.95 is usually indicative of a successful algorithm (109). Recent research has heavily focused on knee segmentation, with Liu et al. designing a model that successfully segmented the different structures of the knee using a CNN combined with a 3D deformable modeling approach (84). Using both T1-rho weighted and 3D double-echo steady-state images, Norman et al. also evaluated a DL model for automated segmentation of knee cartilage and menisci but with simultaneous evaluation of cartilage relaxometry and morphology; they found the model to be adept at generating accurate segmentations and morphologic characterizations when compared to manual segmentations (85). DL techniques can have applications outside the knee as well, as demonstrated by Deniz et al. who used similar methods but shifted attention to the segmentation of the proximal femur, reporting a CNN algorithm with a dice similarity score reaching 0.95 (60). Other venues are also being explored, with AI tools showing promise in neurography segmentation (86) as well as muscle segmentation in osteoarthritis patients to help with muscular trophism evaluation (87).

Besides segmentation, AI may also have applications in radiomics, which is an emerging field in medicine that treats medical images not only as pictures intended solely for visual interpretation but also as a source of diverse quantitative characteristics extracted as mineable data that can be used for pattern identification to eventually assist with decision support, characterization, and prediction of disease processes (110). Spatial distribution of signal intensities and information on pixel interrelationships are mathematically extracted to provide and quantify textural information, which in turn can be used for quantitative imaging biomarker discovery and validation for a number of different conditions such as acute and chronic injuries, spinal abnormalities, and neoplasms (111). By uncovering imperceptible patterns in medical imaging, radiomics-bases predictive models can play different roles such as providing a detailed description of disease burden, identifying relationships between phenotypes and outcomes, and predicting diagnosis and prognosis for certain diseases, ultimately playing a key role in improving precision medicine and personalized patient management (112). ML models can identify and gather imaging characteristics such as the distribution of signal intensities and the spatial relationship of pixels that are not easily discernible with visual interpretation and that can help improve clinical care (113, 114). When testing different ML-augmented radiomics models for preoperative differentiation of sacral chordomas from sacral giant cell tumors on 3D CT; Yin et al. found contrast- enhanced CT features more optimal than non-enhanced features for helping identify the histology of the sacral tumor in question (88). In one retrospective study, Gitto et al. assessed the diagnostic performance of ML-enhanced radiomics-based MRI for the classification and differentiation of atypical lipomatous tumors of the extremities from other benign lipomas, reporting a sensitivity of 92%, a specificity of 33%, and no statistically significant difference when compared to qualitative image assessment performed by a radiologist with 7 years of experience (89). Research into the field is ongoing, and although radiomics has shown promise as a powerful and innovative tool that can help with the evaluation of different types of cancers, more research is needed to fully explore the full scope of its applications (115).

#### Other miscellaneous applications

Several research studies have looked into other potential applications of AI such as joint space evaluation in rheumatoid arthritis (90, 91), epidural mass detection on CT scans (92), rotator cuff pathology detection (116), femoroacetabular impingement and hip dysplasia detection (93), sex determination using CT imaging of the sacrum and coccyx (94) or hand radiographs (95), and assessment of Achilles tendon healing (117). In addition to that, AI applications can have multiple applications in MSK ultrasound (US), including but not limited to segmentation of US images (96), quantitative analysis of skeletal muscles (118), and detection of pediatric conditions such as wrist fractures and developmental dysplasia of the hip (4, 119). Research is still ongoing, and additional repetitive and time-consuming tasks might be tackled in coming years in an attempt to automate more processes and thus accelerate the process of imaging interpretation.

## Results reporting

AI can have several applications that can revolutionize the production of radiology reports and the communication of findings between physicians. Speech recognition, which has already transformed the writing of reports, could be further optimized with DL methods (120). Language processing systems can also be applied, as shown by Do et al. who presented a system capable of recognizing anatomy data from reports generated with speech recognition software to concurrently extract information on possible fractures (121) and Tan et al. who presented a system capable of scanning x-ray and MRI radiology reports to identify lumbar spine imaging findings that could be related to low back pain (122). Natural language processing (NLP) refers to the use of a computer to analyze and interpret human language. Although NLP systems are not entirely novel, recent advances in ML and neural networks have revolutionized this technology, subsequently turning it into a tool that can help with data extraction from radiology reports (123). At their core, NLP systems operate using a multistep approach, beginning with a preprocessing step whereby reports are broken down into different subsets and processing steps during which text from specific sections or differently-weighted sections is split into sentences and words (a process known as tokenization) (124). Word normalization and syntactic analysis follow, whereby spelling mistakes are fixed, medical abbreviations are fully expanded, and word roots are identified with the goal of determining grammatical structures and linking words to semantic concepts (such as symptom or disease), thus assigning meaning to the data (124). The textual features extracted are then processed by an automatic classifier using ML applications to solve the ultimate task assigned to the system (such as information extraction from reports), and ML applications have to be trained on a set of manually-annotated reports, which can be split into a training set and a validation set, both of which are needed to develop the system and assess its performance (124).

Such tools could play a number of roles, such as suggesting management recommendations to radiologists during the dictation of a report or assisting with research purposes by establishing links between different radiological findings and resultant symptomatology or prognosis. Additionally, ML applications may extend to extracting follow-up recommendations from reports, thus ensuring the adequate management of reported key findings (125).

## Limitations

Although AI shows several promising applications across the entire MSK imaging cycle, this technology is still facing a number of challenges and limitations when it comes to both development of AI tools and implementation into clinical practice. Large datasets are needed to develop successful DL tools: tasks or diagnoses for which such datasets are not available might be challenging to automate, and data can be fragmented across many different systems, thus increasing the risk of errors, decreasing the comprehensiveness of datasets, and increasing the expenses of gathering complete data. Moreover, challenges in establishing reference standards, such as irregularities in contouring lesions, diagnostic uncertainties, as well as inconsistencies in human performance and labeling, can all reduce performance and hinder development. DL models being developed are usually trained to perform one single task, whereas patients seen clinically might have a number of etiologies and conditions that require complex simultaneous interpretations.

Given that large amounts of data need to be collected for the development of successful algorithms, issues pertaining to privacy and ownership of such data arise: patients may be concerned that collection of such data is a violation of privacy, especially if an AI model can predict private information about a patient without having received that information and subsequently make it available to third parties (such as life insurance companies). Large datasets can be problematic in a different way: they may be more representative of a specific subset of the population rather than the whole population and could also reflect underlying biases and inequalities in the health system. As such, algorithms trained using such datasets may propagate systemic biases and inequalities that are already present and may not be suitable for treating all patients but rather the subset with the most representation in the training dataset.

Evidently, AI models can and will make mistakes, resulting in errors and injuries to patients being treated using the model. Although medical errors are sometimes inevitable in the medical field and can occur irrespective of the use of AI, the danger of AI-related mistakes is that an underlying problem in one system might result in injuries to thousands of patients if that system becomes widespread (whereas errors from a single human provider will affect the limited number of patients being treated by that provider). Additionally, with errors arises the issue of accountability: models often do not disclose the statistical rationale behind the elaboration of their tasks, making it hard to identify the cause of the error or understand the rationale behind the final output of an algorithm and limiting implementation into medical settings. To catch errors and refine algorithms, post-implementation evaluation, maintenance, and performance monitoring of implemented AI tools is just as vital as pre-implementation development processes to the success of a model. However, such monitoring can prove to be labor-intensive, especially for smaller practices that will inevitably experience workflow disruptions due to a lack of dedicated informatics resources and an increase in the radiologists' burden (126).

## Conclusion

AI, ML and DL have the potential to significantly augment several aspects of the MSK imaging chain, with applications in the ordering of imaging, scheduling, protocoling, acquisition and presentation, image interpretation, as well as report generation and communication of findings. Although research into this technology is showing very promising results, development of tools still faces a number of challenges that impede successful implementation into clinical practice. The ultimate goal is not to design a completely independent system that replaces the need for human expertise but rather to equip radiologists and medical professionals with tools that can automate certain functions and thus alleviate some of the increasing responsibilities radiologists face, affording them more time to focus on more demanding and complex tasks. Radiologists and AI algorithms working hand in hand have the potential to increase the value provided to patients by improving imaging quality and efficiency, patient centricity, and diagnostic accuracy, all of which can greatly enhance both patient and provider satisfaction.
